# Using Plain Language and Adding Communication Technology to an Existing Health-Related Questionnaire to Help Generate Accurate Information: Qualitative Study

**DOI:** 10.2196/jmir.7940

**Published:** 2018-04-23

**Authors:** Marlies Welbie, Harriet Wittink, Marjan J Westerman, Ilse Topper, Josca Snoei, Walter LJM Devillé

**Affiliations:** ^1^ Research Group Lifestyle and Health Faculty of Health Care University of Applied Sciences Utrecht Netherlands; ^2^ Institute of Health Sciences Amsterdam Public Health Research Institute VU University Amsterdam Netherlands; ^3^ Faculty of Social and Behavioral Sciences University of Amsterdam Amsterdam Netherlands; ^4^ Julius Centre for Health Sciences and Primary Care University Medical Centre Utrecht Utrecht Netherlands; ^5^ National Knowledge and Advisory Centre on Migrants Refugees and Health (Pharos) Utrecht Netherlands

**Keywords:** educational status, surveys and questionnaires, physical therapy specialty, qualitative research

## Abstract

**Background:**

Low-educated patients are disadvantaged in using questionnaires within the health care setting because most health-related questionnaires do not take the educational background of patients into account. The Dutch Talking Touch Screen Questionnaire (DTTSQ) was developed in an attempt to meet the needs of low-educated patients by using plain language and adding communication technology to an existing paper-based questionnaire. For physical therapists to use the DTTSQ as part of their intake procedure, it needs to generate accurate information from *all* of their patients, independent of educational level.

**Objective:**

The aim of this study was to get a first impression of the information that is generated by the DTTSQ. To achieve this goal, response processes of physical therapy patients with diverse levels of education were analyzed.

**Methods:**

The qualitative Three-Step Test-Interview method was used to collect observational data on actual response behavior of 24 physical therapy patients with diverse levels of education. The interviews included both think-aloud and retrospective probing techniques.

**Results:**

Of the 24 respondents, 20 encountered one or more problems during their response process. The use of plain language and information and communication technology (ICT) appeared to have a positive effect on the comprehensibility of the DTTSQ. However, it also had some negative effects on the interpretation, retrieval, judgment, and response selection within the response processes of the participants in this study. No educational group in this research population stood out from the rest in the kind or number of problems that arose. All respondents recognized themselves in the outcomes of the questionnaire.

**Conclusions:**

The use of plain language and ICT within the DTTSQ had both positive and negative effects on the response processes of its target population. The results of this study emphasize the importance of earlier recommendations to accompany any adaption of any questionnaire to a new mode of delivery by demonstrating the difference and equivalence between the two different modes and to scientifically evaluate the applicability of the newly developed mode of the questionnaire in its intended setting. This is especially important in a digital era in which the use of plain language within health care is increasingly being advocated.

## Introduction

### Background

It is widely known and accepted that patient-centered care has the potential to increase the effectiveness of health care in general [1]. Unfortunately low-educated patients are not always able to benefit from a patient-centered care approach. A possible explanation for this can be found in the fact that patient-centered care demands of patients to take an active mutual partnership in the patient-provider interaction [2,3]. Patient-centered care puts a relatively strong emphasis on communication and information and takes the patient’s perspective as a starting point [4,5]. Low-educated people have trouble providing information about their health problems to health care professionals. It is often hard for them to determine which information their health care provider (HCP) needs. The majority of them lack the health care vocabulary to report symptoms accurately, and they tend to provide information in a way that is illogical and difficult to comprehend by their HCP [6]. Having trouble providing information causes problems in patient-provider interaction, which impacts health outcomes negatively [7]. Evidence shows that the use of standardized health-related questionnaires contributes to the quality and patient centeredness of patient-provider interaction [8-13]. However, as most health-related questionnaires are not designed in ways that meet the needs of low-educated patients, these patients are disadvantaged in using them effectively within the health care setting [11-13]. In 2016, 9.5% of the Dutch population in the age range of 15 to 75 years had an educational level of primary school at most [14]. These people specifically are at risk when it comes to understanding and using health information [15]. If low-educated patients would be able to complete standardized health-related questionnaires independently and accurately, this might help them to provide relevant information on their health problem in a way that is logical and understandable to their HCP.

The content of the most frequently used questionnaire in Dutch physical therapy practice [16], the Patient Specific-Complaint (PSC) questionnaire [17], fits the goal of helping patients to provide relevant information regarding their health problem to their physical therapist. It is aimed at making the patient select his main limitations in functioning and formulate his own specific treatment goals. This paper-based questionnaire is responsive and sensitive to change to complaints that are highly relevant to the individual patient [17,18]. However, all members of the Dutch study sample of a recent study on the PSC questionnaire had problems completing it independently. All these 25 respondents, whose education levels varied from primary education to doctoral degrees, had trouble comprehending and interpreting one or more parts of the questionnaire. Six of them had difficulties finding a well-fitting answer to one or more questions. Due to these problems, the questionnaire generated invalid information in thirteen cases. Within the group respondents who provided invalid information, patients with no or primary education only were overrepresented [19].

The Dutch PSC questionnaire [17] was used as a starting point for the development of a user-friendly health-related questionnaire that meets the needs of low-educated physical therapy patients. This aim was met by using plain language and taking advantage of the possibilities of information and communication technology (ICT) by offering alternatives to text (eg, audio, pictures, and movies), self-explanatory scales, and easily accessible background information on the questionnaire’s rationale. This resulted in the prototype of a new interactive questionnaire called the Dutch Talking Touch Screen Questionnaire (DTTSQ). The codesign process that led to the development of this prototype was described in detail by Cremers et al in 2015 [20].

### Objective

The aim of this study was to get a first impression of the validity of the prototype of the DTTSQ by analyzing the response processes of patients with diverse levels of education. The research question that underlay this study was, “What problems occur during the response process of physical therapy patients with diverse levels of education while they complete the Dutch Talking Touchscreen Questionnaire?”

## Methods

### Design

A qualitative study was conducted. The Three-Step Test-Interview (TSTI) method [21] was used to collect observational data on actual response behavior of the respondents. The interviews included both think-aloud and retrospective probing techniques. Qualitative pretesting of questionnaires using cognitive methods such as the TSTI [21] is a well-known step within the development process of health-related questionnaires [22-25]. It enables researchers to give answers to questions such as the following: do all respondents understand the questions in the same way, do the questions ask for information that the respondents have and can retrieve, and does the wording of the questions provide respondents with all necessary information they require to be able to answer them in the way that was intended by its developers [22]?

### Device

The DTTSQ was developed during a user-centered design process [20], which meant that low-educated persons were closely involved in designing the questionnaire. As a result, questions about pain location and pain intensity were added to the original questions that addressed the nature and severity of limitations in activities of daily living and the priority in which these limitations should be focused on during physical therapy. Needs regarding ease of use were met by the use of visual (pictures and videos) and auditory (speech) support, which was added to the questions. Respondents could insert their answers by tapping on the touch screen. The DTTSQ started with an introductory video clip in which a host explains the purpose of the questionnaire and gives instructions on how to use the questionnaire (see Multimedia Appendix 1, screenshot 1 “Welcome”). All the questions were shown on separate screens. The application did not have a back function, so respondents could only move forward. After the first three questions, a new clip was shown to introduce and operationalize the term “activities” and to give instructions on an additional navigation function within the activity screens (see Multimedia Appendix 1, screenshot 6 “Activities”). The questionnaire finished with a video clip in which the host thanked the respondent for completing the questionnaire, explained what the physical therapist would do next, and announced that the questionnaire would end and close down automatically (Multimedia Appendix 1, screenshot 16 “Thank you”). To help patients keeping track of their answering process, overviews of their answers were shown regularly during the response process (see Multimedia Appendix 1; screenshot 5 “Overview location of the health problems,” screenshot 8 “Overview activities,” screenshot 10 “Overview most important activities,” screenshot 14 “Overview most important activities and effort,” and screenshot 15 “Overview all outcomes of the questionnaire”). For respondents who needed help or wanted more information on questions and/or answering options, a help function was provided. When the help function was activated, the question and answering options, as well as instructions on operation and background information on the questions, were given in spoken word [20].

### Recruitment Strategy and Participants

Recruitment took place in eleven primary care practices in deprived areas of Utrecht, The Netherlands. Potential participants were invited by their physical therapists to participate in this study. The physical therapists shortly explained the goal of the study and provided the patient with an information letter that was written in plain Dutch language. If patients were interested, the physical therapist asked permission to give the patients’ telephone number to researcher IT. Then researcher IT (1) Contacted the patient by telephone, (2) Again shortly explained the aim of the study, (3) Made sure the patient understood what was asked of him/her, (4) Answered any question the potential participant may have had, and (5) Checked the inclusion criteria. Inclusion criteria for participants were as follows: aged 18 years or older, Dutch as their first language, and both parents born in The Netherlands. The sampling procedure was aimed at getting a broad variation in levels of education and age, plus balance in our sample regarding gender. Throughout the recruitment process, the recruiting physical therapists were constantly kept informed about the profiles of participants the researchers were looking for. In total, 24 physical therapy patients were included in this study. Characteristics of study population can be found in Tables 1 and 2.

### Data Collection and Procedures

Data collection took place at the respondents’ homes or at the physical therapy practice of the respondent’s physical therapist. The choice of location depended on the preference of the respondent. Two researchers were present (researchers IT and JS). Researcher IT conducted the interviews. When researcher JS missed information, she asked complementary questions.

The TSTI method was conducted as follows [21]:

#### Step 1

Researchers IT and JS observed each respondent as they completed the DTTSQ while thinking out loud. This step was aimed at collecting observational data regarding the respondent’s response behavior. The data collected consists of two types: (1) observations of respondent’s behavior and (2) think-aloud data. The data were recorded in the form of videotapes as well as audiotapes for later analysis and real-time notes by the researchers for use during the interview itself and later analysis. The researchers wrote their notes down on hardcopies of print screens of the DTTSQ.

#### Step 2

After the respondent finished completing the DTTSQ, researcher IT conducted an in-depth interview to clarify and complete the observational data. During this step, researcher IT only focused on those actions or thoughts she felt not fully informed about or were not fully clear to her. This step was aimed at filling gaps in the observational data and check information.

#### Step 3

During the final step, researcher IT conducted a semistructured interview aimed at eliciting experiences and opinions of the respondent. In this part of the interview, the respondent was stimulated to add secondary data such as accounts and reports of feelings, explanations, preferences, recommendations, etc. Researcher IT asked the respondent to paraphrase questions and to explain in his own words how he interpreted the question and why he chose the answering options he chose. When a respondent encountered problems in responding to a question, he was asked what he thought the exact nature of the problem was and why he behaved as he did in response to the question. He also was asked for suggestions for improvement of the question in terms of wording, layout, instructions, etc. Additionally, the respondent was asked to describe his health problem(s) and treatment goal(s) in his own words. Comparing these descriptions to the respondent’s responses to the questionnaire during step 1 of the TSTI provided useful information as indicators of the validity of the data collected by the DTTSQ. Finally, the respondent was asked if he recognized himself in the outcomes of the questionnaire that were shown at the end of the questionnaire (see Multimedia Appendix 1, screenshot 15 “Overview all outcomes of the questionnaire”). After the TSTI was finished, researcher IT collected the demographic data through a brief structured interview.

**Table 1:** Characteristics of the respondents subdivided according to their level of education.

Characteristic		Low-educated^a^ respondents (n=6)	Moderately educated^b^ respondents (n=13)	Highly educated^c^ respondents (n=5)
Mean age (range), years		65.8 (47-79)	50.5 (18-73)	56 (32-76)
**Gender, n**				
	Male	2	5	2
	Female	4	8	3

^a^Low means no education or primary education.

^b^Moderately means lower secondary education, (upper) secondary education, or postsecondary nontertiary education (including vocational education).

^c^Highly means tertiary education (bachelor’s degree or higher).

**Table 2:** Characteristics per respondent.

Pseudonym	Age (year)	Educational level^a^	Last occupation
Jerome	47	Low	Truck driver
Michelle	56	Low	Cleaning lady
Ida	66	Low	Cleaning lady
Ronald	70	Low	Home painter
Dora	77	Low	Cleaning lady
Ilene	79	Low	Cleaning lady
Peter	18	Moderate	Student
Jude	18	Moderate	Student
Joline	19	Moderate	Photographer
Sandra	39	Moderate	Graphic designer
Christine	39	Moderate	Nurse for mentally disabled people
Lydia	56	Moderate	Domiciliary care
Rose	60	Moderate	Saleswoman
Francine	61	Moderate	Administrative officer
Henry	64	Moderate	Project coordinator
Bob	68	Moderate	Cashier
Roger	70	Moderate	Home painter
Bill	72	Moderate	Order picker
Mia	73	Moderate	Administrative officer
Ellen	32	High	Management assistant
Helga	54	High	Artist
Jill	55	High	Management assistant
Harald	63	High	Financial controller
Bernie	76	High	Lecturer chemistry

^a^Low refers to no education or primary education; moderate refers to lower secondary education, (upper) secondary education, or postsecondary nontertiary education (including vocational education); and high refers to tertiary education (bachelor’s degree or higher).

### Data Analysis

Data were analyzed using a thematic content analysis approach [26]. Four types of data were analyzed: (1) video recordings of the first two steps of the interview, (2) Dutch transcriptions of the third step of the interview, (3) observed respondent behavior in field notes, and (4) background information regarding the educational level, age, gender, and occupation of each respondent. Researcher MW started with open coding, coding all fragments of the twenty-four transcripts of step three of each interview using MAXQDA 10 of VERBI Software GmbH, Berlin.

The codes and fragments of seven randomly selected transcripts were validated by two peer researchers by independently coding each transcript with the coding scheme developed by researcher MW. Differences in fragmentation or coding were discussed during consensus meetings.

To get more familiar with the data and to create an overview, researcher MW made a descriptive summary of each case on the basis of all four types of generated data after she finished open coding. Each summary contained all emerging themes regarding problems that occurred during the four phases of the response process as described by Tourangeau: (1) *comprehension*: (a) comprehension of text and wording and (b) interpretation of the meaning of the text, (2) *retrieval*: gathering relevant information, (3) *judgment*: assessing the retrieved information to judge its adequacy in relation to the meaning of the question, and (4) *response selection*: selecting the best fitting answering option [27]. The emerged themes in the summaries were supplemented with related field notes and background information regarding the educational level, age, gender, and occupation of the respondent. Then researcher MW listed all emerging “themes” from the descriptive summaries regarding problems that arose during the four steps of the response process. She established which themes recurred or were common and which were less common or stood alone. Then she structured the earlier created coding scheme by arranging all open codes by labeling them as, “problem with comprehension,” “problem with interpretation,” “problem with retrieval,” “problem with judgment,” or “problem with response selection.”

The following step in analyzing the data was comparing the description of the limitations in functioning and treatment goals described by respondents during the semistructured interview (interview step 3) to the answering options the respondent selected in the DTTSQ during the think-aloud phase of the data collection (interview step 1). If the chosen answer during step 1 did not fit the description in step 3, researcher MW closely watched the video again to see which actions or thoughts during the four steps of Tourangeau [27] during the response process of the question led the respondent to select the chosen answering option.

As a last step, researcher MW compared the analyzed interviews of low, moderately, and highly educated respondents to see whether or not the problems that occurred during the response processes differed between these groups of respondents. Transcripts were made in Dutch language. Only quotes used in this paper were translated from Dutch to English by researcher MW and checked by researcher HW, who is a bilingual speaker. During the whole course of the study, procedures and results were checked and discussed with researchers HW, MJW, and WD.

### Ethics

No external funding was received by the Utrecht University of Applied Sciences to conduct this study. The study was registered with the Medical Ethics Commity of the Acadamic Medical Centre of Amsterdam, which declared that it does not fall under the scope of the “Medical Research Involving Human Subjects Act.” The study was conducted according to the principles of the Declaration of Helsinki. All respondents provided written informed consent. The respondents names used in this paper are all fictitious to protect their privacy.

## Results

### Encountered Problems

Of the 24 respondents, 20 encountered one or more problems during their response process. Low-educated Michelle and moderately educated Christine, Lydia, and Sandra did not encounter any problem. All members of the total study population stated that they recognized themselves in the overall outcomes of the questionnaire. Bernie stated:

If I would have developed this questionnaire so it would have fitted my health problem I would have done it differently. Instead of selecting specific points on the body chart, for instance, I would have enabled people to select regions. In my case that would have enabled me to select the whole lower part of my body instead of a few specific points in it. But even though I would have done it differently, I recognize myself in the summary of my limitations in functioning. That is mainly due to the pictures of the activities in which I am impaired. When I look at all the outcomes as a whole, it is right. I recognize my own health situation.

Most problems concerned interpretation of questions and answering options. Questions 1 and 4 generated the most problems. Question 3 generated no problems at all (see Table 3).

### Problems With Comprehension of Text and Wording, and Interpretation

There were no problems with comprehension of text and wording. A total of 13 respondents of all educational groups encountered problems with interpretation. Ronald and Bob encountered this problem with three questions and Helga and Jerome with two different questions. The other 9 respondents encountered this problem with one question.

**Table 3:** Number of respondents having problems per question for each step of the response process. Hyphen indicates nonapplicabilty.

Question or assignment	Comprehension problems	Interpretation problems	Retrieval problems	Judgment problems	Response selection problems
1. Do you have pain? (Multimedia Appendix 1: screenshot 2 “Pain”)	-	6	-	-	-
2. Tap on the location of your health problem. You can tap on multiple locations. (Multimedia Appendix 1: screenshot 3 “Location of the health problem”)	-	-	1	1	2
3. This is the location of your pain. Rate the severity of your pain on the scale below. (Multimedia Appendix 1: screenshot 4 “Pain severity”)	-	-	-	-	-
4. Select the activities in which you are impaired. (Multimedia Appendix 1: screenshot 7 “Activity “Lying”)	-	9	6	8	3
5. Select the three activities which are most important to you. (Multimedia Appendix 1: screenshot 9 “most important activities”)	-	-	-	-	2
6. Select the activity which is most important to you. (Multimedia Appendix 1: screenshot 11 “Most important activity 1”)	-	1	-	-	-
7. Which of these two activities is most important to you now? (Multimedia Appendix 1: screenshot 12 “Most important activity 2”)	-	1	-	-	-
8. Rate the effort it takes to carry out this activity. (Multimedia Appendix 1: screenshot 13 “Effort activity 1”)	-	2	-	-	2

#### Interpreting Pictures

**Figure 1:**
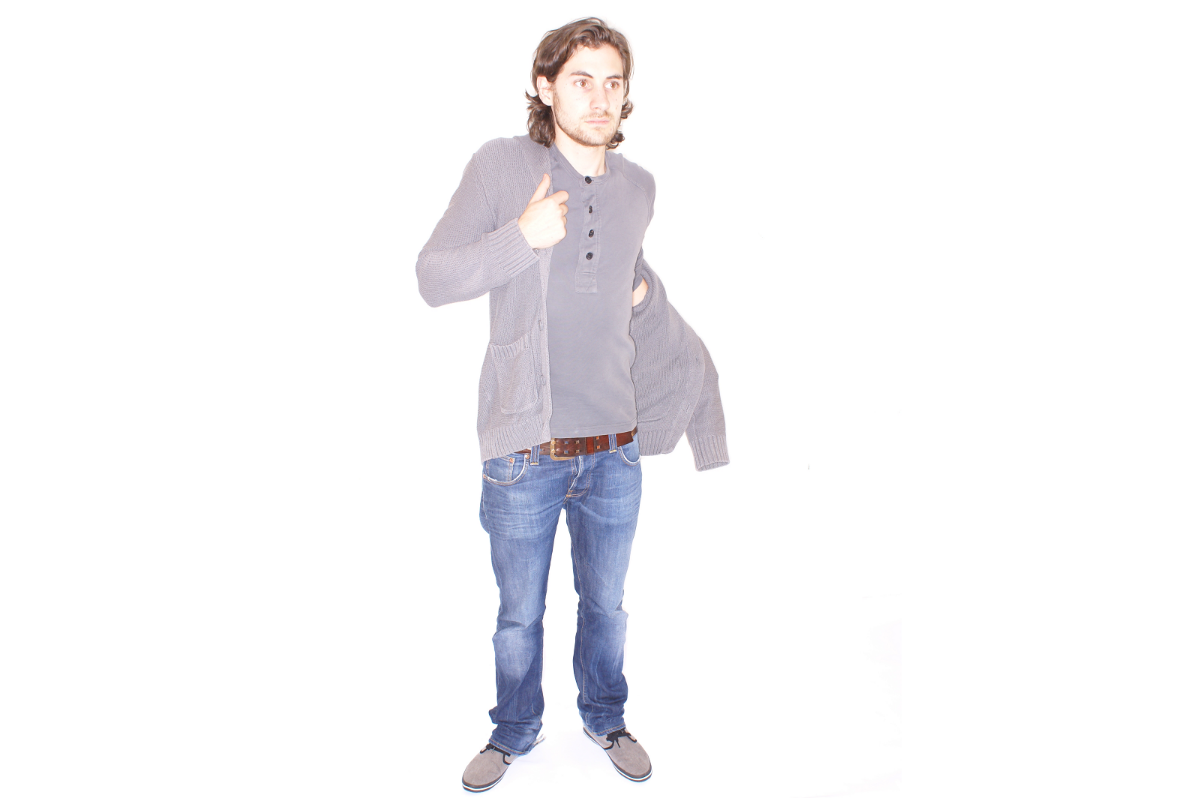
Activity: "dressing and undressing".

**Figure 2:**
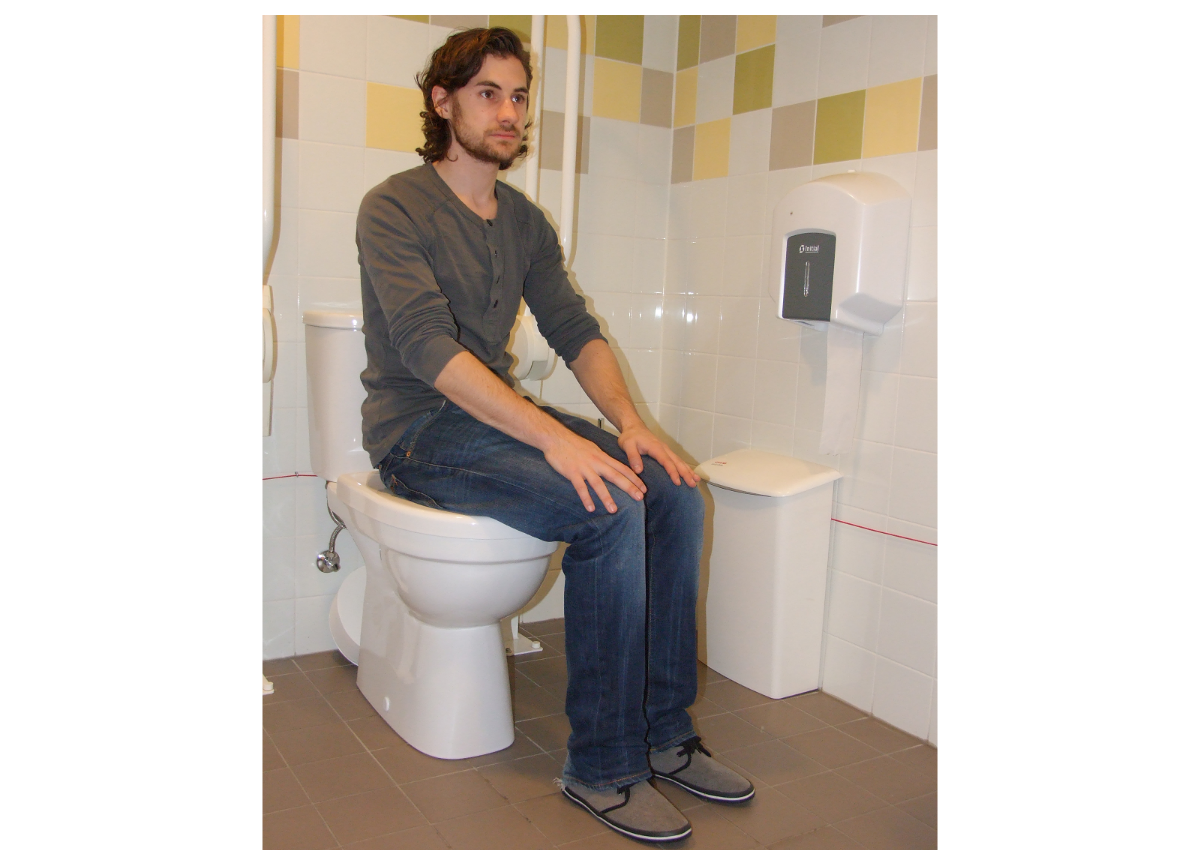
Activity: "going to the toilet".

A total of 7 respondents interpreted pictures that were used as answering options in question 4 differently than was intended by the developers of the questionnaire. Ilene, for instance, selected “dressing and undressing” (Figure 1) and going to the toilet (Figure 2) because the way in which the person in the picture carried out the activity and the context in which he did it were different from theirs. This is illustrated in the following conversation:

Ilene:

I selected “dressing and undressing” because the person on the photo is standing up while he is dressing himself. I cannot do that. I have to sit down.

Interviewer:

Would you have selected this activity if the person on the photo was sitting down while he dressed and undressed himself?

Ilene:

No, because that is no problem for me. That is the way I do it. It is the same with going to the toilet. I selected that photo because the person on the photo does not use the support arms while he is using the toilet.

Interviewer:

Would you have selected the photo if he would have used the support arms?

Ilene:

No of course not! I do not have any problem going to the toilet because I have these support arms. I have everything I need in my house.

#### Interpreting Categories

The answering options in the form of pictures of question 4 were put into eight different activity categories. These categories were shown on eight separate screens. The use of categories influenced the response process of two respondents negatively. Rose, for instance, recognized her impairment in the activity “reaching for something above the head,” but did not select it:

I really was in great doubt with “reaching!” Because I thought: yes indeed that is problematic for my shoulder so I should select that activity. But the activity was placed in the category “standing” which I associated with using the legs and back, not with arm movements. In hindsight I probably should have selected it, but when I was completing the questionnaire I chose not to.

#### Interpreting Plain Language

Six respondents misinterpreted question 2 “Do you have pain?” Four of them mentioned the short and simple way in which the question was formulated as the reason for this misinterpretation. All six respondents selected the answer “no,” whereas in fact they were seeking help with their physical therapist because of pain complaints. Henry stated:

Well I am not in pain at this moment. But when I go photographing I take long walks carrying heavy lenses. And then my hip hurts sometimes. This is something my physical therapist needs to know because it should be the aim of the treatment. But I interpreted the question as “are you in pain at this moment.” And that is why I answered “no.” The sentence, the question, is very short. It is not specific enough. It should have said: “Are you in pain during certain activities” or something.

#### Differences in Professional and Layman Interpretations

Although “getting up and sitting down” and “getting in and out of a car” are different activities from a physical therapist’s perspective, these are very similar movements from the perspective of moderately educated Bob, who stated:

Well “getting up and sitting down” and “getting in and out of a car” are kind of the same activities to me. So it is hard for me to say which one is more important in answer to question 6 and 7. I know I selected “getting in and out of the car” as the most important activity when I was completing the questionnaire. But when I would have to choose again I would go with “getting up and sitting down,” because that is more generic and therefore it occurs more frequently in daily life.

#### Interpreting the Numeric Rating Scale

Low-educated Dora and Ronald scored the numeric rating scale of question 8 backwards. They interpreted 10 as “no effort” and 0 as “the most effort possible.”

### Problems With Retrieval

A total of 7 respondents of all educational groups had problems retrieving information during their response processes.

#### Lack of Retrieval Because of the Form of Answering Options

A total of 4 respondents did not retrieve information because of the lack of answering options. They looked at the body chart of question 2 and the pictures of question 4 and searched their memory for any health problems related to the answering options. As a result, existing health problems that were *not* associated with the given answering options were *not* retrieved from memory. After Harald finished completing the questionnaire, he told the researcher that he was impaired in pulling objects, which is not a given answering option in question 4. Harald stated:

I did not miss it while I was completing the questionnaire. I probably thought that that picture would come later or in another category or something. I don’t know. I did not really notice that it wasn’t there.

#### Lack of Retrieval Because of Memory Issues

In three cases, the root of the problem seemed to be a memory issue, which was not related to the content or form of the questionnaire. Ellen, for instance, described to the interviewer why she selected the activities “lifting” and “carrying” in answer to question 4. During this description, her recollection of the health problem became clearer. This made her realize in hindsight that “picking something up from the floor” would have been a better answer.

### Problems With Judgment

A total of 9 respondents of all educational levels encountered problems with judgment.

#### Retrieved Information Judged as “Adequate to Answer the Question” Was Not Related to Physical Therapy (Anymore)

All 8 respondents indicated health problems that were not part of their treatment goal for physical therapy (anymore). Bob, for instance, indicated on the body chart that he had pain in his neck and shoulders, *and* he had low back pain. During the interview, he told the researcher that his neck and shoulder pain were chronic and existed for many years now. He did not believe it would be of any use for the physical therapist to put effort into trying to ease this pain. Therefore, it was not a part of his treatment goals. He was seeking help from his physical therapist for his acute low back pain.

### Problems With Response Selection

A total of 8 respondents of all educational levels had problems with response selection. Bernie encountered this problem with two different questions of the questionnaire.

#### Not Able to Select the Right Answering Option Because These Options Do Not Match the Respondent’s Response to the Question

All 8 respondents had problems with response selection because the response items did not match their answer(s). Bernie for instance had a complaint that was not “touchable” or located at a particular part of the body. But he was forced to place a dot on the body chart to be able to go on to the next question. Bernie stated:

This is not right at all! It says: “tap on the location of your health problem.” But then one has to be able to locate his complaints. I can’t. The way I walk does not feel normal to me, it does not feel the way it used to feel. I cannot say that I feel it “in my legs.” It really is the movement itself that feels “off.” I go to the physical therapist to find out what causes this. So at this moment I don’t know where the root of the problem is located. Because I am forced to point out a location and the legs are clearly involved in walking, I have put a dot on the legs. But it is just not right. I mean, when I would have had pain in my hand I could have answered this question. If I would have felt it in my foot I would have tapped on the foot. But in my case it is about the movement...

## Discussion

### Principal Findings

Of the 24 respondents, 20 encountered one or more problems during their response process. No problems were experienced with comprehension of text or wording. Most problems arose with (1) Interpretation of pictures and plain language, (2) Respondents not retrieving health problems that were *not* associated with the given answering options, and (3) Respondents judging retrieved health problems as relevant, although these were not related to their physical therapy treatment goals. No educational group in this research population stood out from the rest in the kind or number of problems that arose.

Despite the fact that 20 respondents did not respond to each question in the way that was intended by its developers, all respondents recognized themselves in the outcomes of the questionnaire shown in a screen summary.

### Comparison With Prior Work

The clarity of text and wording seems to be better in the DTTSQ than in the PSC questionnaire [17], which was used as a starting point for development. In the study on the response process of the PSC questionnaire, “comprehension” and “interpretation” were put together into one category called “problems with reading and comprehending the questionnaire” [19]. Due to the way in which the data was collected in the PSC questionnaire study (lacking a think-aloud component), even in hindsight it is not always possible to determine if the source of each “problem with reading and comprehending the questionnaire” was comprehension or interpretation. This makes the PSC questionnaire and DTTSQ studies not fully comparable in this respect. Still, little over half of the respondents in the DTTSQ study versus all respondents in the PSC questionnaire study had comprehension and/or interpretation problems.

Invalid answers were reported in 52% (13/25) of the Dutch subjects in the PSC questionnaire study [19]. In this study, the percentage of respondents that gave one or more invalid answers was much higher: 83% (20/24) cases. Again the data of these two studies are not fully comparable. The PSC questionnaire study did not contain a think-aloud component. Having a think-aloud component in a study tends to add data on validity of answers, while at the same time there is no loss of data in comparison to studies without a think-aloud component [21]. This may be an explanation for the considerable difference between the amount of invalid answers found between the two studies.

Except for the problems caused by the use of plain language, using pictures as answering options and showing questions on separate screens without a back function, the problems found in this study were not new or exclusive for the DTTSQ. Problems such as “differences in layman and professional perspective” and “memory issues” are commonly seen in comparable studies and well documented in Tourangeau’s book “The Psychology of Survey Response” [23,24,27,28].

### Problems Caused by the Use of Plain Language

Four out of 6 respondents that misinterpreted question 1 of the DTTSQ “Do you have pain” mentioned the short and simple formulation of this question as the root of the problem. The formulation of question 1 and the layout of the screen on which it was shown was in line with the “European Easy-to-Read Guidelines” [29]. With the formulation of this question, however, the developers of the questionnaire may not have done enough justice to the complex concept of pain. It may be necessary to provide more detailed background information on the purpose and focus of the question [30]. Considering that understanding *spoken* language is easier to people than understanding *written* language [31], it might be recommended to add information by using a voice-over. In this way, information on the purpose and focus of the question and/or answering option(s) can be given without making the reading task more difficult [32].

### Problems Caused by the Design of the User Interface

#### Use of Pictures

In addition to plain language, pictures were used to contribute to the comprehensibility of the questionnaire. Respondents’ interpretation of the pictures did not always match the intended meaning by its developer. Optimizing this match by testing the interpretation of newly developed pictures in the target population before they are used in the questionnaire is recommended during the further development of the DTTSQ.

#### Showing All Questions on Separate Screens

The questions of the DTTSQ were shown in separate screens, and respondents were not able to go back to earlier screens. This makes the response process different from that of paper-based questionnaires in which respondents are able to oversee the whole questionnaire, choose the order in which they answer questions, and go back and forth between questions. The answering options of question 4 were subdivided into eight categories shown on eight separate screens. Lacking the complete overview of all answering options may have complicated the decision on whether or not to select an activity because the respondent was not able to see whether or not pictures in coming screens would be a better fit. Giving a complete overview of all answering options, for instance by presenting them as thumbnail images [33] and providing a back option, may help to reduce the amount of problems with response selection.

### Limitations

This study was not designed to reach data saturation. The goal was to get a first impression of the response processes of respondents with diverse educational levels completing the prototype of the questionnaire to be able to make informed choices in further development of the questionnaire. Because twenty-four cases were included in this study, it can be assumed that the most common problems have been exposed [34].

### Conclusions

The use of plain language and ICT within the DTTSQ has had positive and negative influences on the response processes of the research population.

Results of recent reviews and articles on the comparability of paper-based and electronic versions of questionnaires may give the impression that digitalizing questionnaires can be done without influencing psychometric properties [35-39] and response rates [40-44]. This is true when the digital version is a near copy of the paper-based questionnaire in terms of content and layout. But in an era in which the use of plain language and “inclusive design” or “electronic health for all” [45,46] is being advocated increasingly [47,48], copying the content and layout of the original into the digital version may not be enough.

The results of this study emphasize the importance of two basic recommendations, which are as follows: (1) accompany any adaption of any questionnaire to a new mode of delivery by evidence, demonstrating the difference and equivalence between the two different modes [49] and (2) scientifically evaluate the applicability of the newly developed mode of the questionnaire in its intended setting, to assess if it meets the standard criteria of validity, reproducibility, and feasibility [50]. Such studies should be designed and executed in a way that suits the (in)abilities of the target population of the questionnaire that is being evaluated. Like the qualitative method chosen in this study suited the (in)abilities of low-educated and/or low-literate participants by not demanding any reading or writing skills from study participants.

## Appendix

Multimedia Appendix 1 An overview of the screenshots of the Dutch Talking Touch Screen Questionnaire.

## Abbreviations

DTTSQ: Dutch Talking Touch Screen Questionnaire
HCP: health care provider
ICT: information and communication technology
PSC: Patient Specific-Complaint
TSTI: Three-Step Test-Interview

## References

[1] Berwick DM, Nolan TW, Whittington J (2008). The triple aim: care, health, and cost. Health Aff (Millwood).

[2] de Boer D, Delnoij D, Rademakers J (2013). The importance of patient-centered care for various patient groups. Patient Educ Couns.

[3] Saha Somnath, Beach Mary Catherine (2011). The impact of patient-centered communication on patients' decision making and evaluations of physicians: a randomized study using video vignettes. Patient Educ Couns.

[4] Bensing J (2000). Bridging the gap. The separate worlds of evidence-based medicine and patient-centered medicine. Patient Educ Couns.

[5] Mead N, Bower P (2000). Patient-centredness: a conceptual framework and review of the empirical literature. Soc Sci Med.

[6] Williams MV, Davis T, Parker RM, Weiss BD (2002). The role of health literacy in patient-physician communication. Fam Med.

[7] Paasche-Orlow MK, Wolf MS (2007). The causal pathways linking health literacy to health outcomes. Am J Health Behav.

[8] Nelson EC, Hvitfeldt H, Reid R, Grossman D, Lindblad S, Mastanduno MP, Weiss LT, Fisher ES, Weinstein JN (2012). Using Patient-Reported Information to Improve Health Outcomes and Health Care Value: Case studies from Dartmouth, Karolinska and Group Health.

[9] Calvert M, Brundage M, Jacobsen PB, Schünemann HJ, Efficace F (2013). The CONSORT patient-reported outcome (PRO) extension: implications for clinical trials and practice. Health Qual Life Outcomes.

[10] Kotronoulas G, Kearney N, Maguire R, Harrow A, Di DD, Croy S, MacGillivray S (2014). What is the value of the routine use of patient-reported outcome measures toward improvement of patient outcomes, processes of care, and health service outcomes in cancer care? A systematic review of controlled trials. J Clin Oncol.

[11] Hahn EA, Cella D (2003). Health outcomes assessment in vulnerable populations: measurement challenges and recommendations. Arch Phys Med Rehabil.

[12] Paiva CE, Siquelli FA, Zaia GR, de Andrade DA, Borges MA, Jácome AA, Giroldo GA, Santos HA, Hahn EA, Uemura G, Paiva BS (2016). Development of a new multimedia instrument to measure cancer-specific quality of life in Portuguese-speaking patients with varying literacy skills. Springer Plus.

[13] El-Daly I, Ibraheim H, Rajakulendran K, Culpan P, Bates P (2016). Are patient-reported outcome measures in orthopaedics easily read by patients?. Clin Orthop Relat Res.

[14] Onderwijsincijfers.

[15] van der Heide I, Wang J, Droomers M, Spreeuwenberg P, Rademakers J, Uiters E (2013). The relationship between health, education, and health literacy: results from the Dutch adult literacy and life skills survey. J Health Commun.

[16] Swinkels RAHM, van Peppen RP, Wittink H, Custers JWH, Beurskens AJHM (2011). Current use and barriers and facilitators for implementation of standardised measures in physical therapy in the Netherlands. BMC Musculoskelet Disord.

[17] Beurskens AJ, de Vet HC, Köke AJ, Lindeman E, van der Heijden GJ, Regtop W, Knipschild PG (1999). A patient-specific approach for measuring functional status in low back pain. J Manipulative Physiol Ther.

[18] Beurskens AJ, de Vet HC, Köke AJ (1996). Responsiveness of functional status in low back pain: a comparison of different instruments. Pain.

[19] Welbie M, Wittink H, Westerman M, Devillé W (2016). Completing the patient specific-complaint questionnaire in physical therapy practice is problematic for high and low literate patients: a qualitative study. Int J Pers Cent Med.

[20] Cremers AH, Welbie M, Kranenborg K, Wittink H (2015). Deriving guidelines for designing interactive questionnaires for low-literate persons: development of a health assessment questionnaire. Univ Access Inf Soc.

[21] Hak T, van der Veer K, Jansen H (2008). The three-step test-interview (TSTI): an observation-based method for pretesting self-completion questionnaires. Surv Res Methods.

[22] Collins D (2003). Pretesting survey instruments: an overview of cognitive methods. Qual Life Res.

[23] ten Velden M, Couldrick L, Kinébanian A, Sadlo G (2012). Dutch children's perspectives on the constructs of the child occupational self-assessment (COSA). OTJR.

[24] Liu RD, Buffart LM, Kersten MJ, Spiering M, Brug J, van Mechelen W, Chinapaw MJ (2011). Psychometric properties of two physical activity questionnaires, the AQuAA and the PASE, in cancer patients. BMC Med Res Methodol.

[25] Johnson C, Aaronson N, Blazeby JM, Bottomley A, Fayers P, Koller M, Kulis D, Ramage J, Sprangers M, Velikova G, Young T (2011). EORTC.

[26] Braun V, Clarke V (2006). Using thematic analysis in psychology. Quol Res Psychol.

[27] Tourangeau R, Rasinski K (2000). The psychology of survey response.

[28] Pool JJM, Hiralal SR, Ostelo RWJG, van der Veer K, de Vet HC (2010). Added value of qualitative studies in the development of health related patient reported outcomes such as the Pain Coping and Cognition List in patients with sub-acute neck pain. Man Ther.

[29] Freyhoff G, Hess G, Kerr L, Menzel E, Tronbacke B, Van der Veken K (1998). ILR.

[30] (2014). MSKTC.

[31] Sousa DA (2014). How the brain learns to read.

[32] Johnson J (2014). Designing with the mind in mind.

[33] Johnson J, Roberts T, Verplank W, Smith D, Irby C, Beard M, Mackey K (1989). The Xerox star: a retrospective. Computer.

[34] Nielsen J, Landauer TK (1993). A mathematical model of the finding of usability problems. http://portal.acm.org/citation.cfm?doid=169059.169166.

[35] Norquist J, Chirovsky D, Munshi T, Tolley C, Panter C, Gater A (2017). Assessing the comparability of paper and electronic versions of the EORTC QOL module for head and neck cancer: a qualitative study. JMIR Cancer.

[36] Lundy JJ, Coons SJ, Aaronson NK (2014). Testing the measurement equivalence of paper and interactive voice response system versions of the EORTC QLQ-C30. Qual Life Res.

[37] Campbell N, Ali F, Finlay AY, Salek SS (2015). Equivalence of electronic and paper-based patient-reported outcome measures. Qual Life Res.

[38] Muehlhausen W, Doll H, Quadri N, Fordham B, O'Donohoe P, Dogar N (2015). Equivalence of electronic and paper administration of patient-reported outcome measures: a systematic review and meta-analysis of studies conducted between 2007 and 2013. Health Qual Life Outcomes.

[39] Gwaltney CJ, Shields AL, Shiffman S (2008). Equivalence of electronic and paper-and-pencil administration of patient-reported outcome measures: a meta-analytic review. Value Health.

[40] Horevoorts NJ, Vissers PA, Mols F, Thong MS, van de Poll-Franse LV (2015). Response rates for patient-reported outcomes using web-based versus paper questionnaires: comparison of two invitational methods in older colorectal cancer patients. J Med Internet Res.

[41] van den Berg MH, Overbeek A, van der Pal HJ, Versluys AB, Bresters D, van Leeuwen FE, Lambalk CB, Kaspers GJL, van Dulmen-den Broeder E (2011). Using web-based and paper-based questionnaires for collecting data on fertility issues among female childhood cancer survivors: differences in response characteristics. J Med Internet Res.

[42] Kilsdonk E, van Dulmen-den Broeder E, van der Pal HJ, Hollema N, Kremer LC, van den Heuvel-Eibrink MM, van Leeuwen FE, Jaspers MW, van den Berg MH (2015). Effect of web-based versus paper-based questionnaires and follow-up strategies on participation rates of Dutch childhood cancer survivors: a randomized controlled trial. JMIR Cancer.

[43] Duracinsky M, Lalanne C, Goujard C, Herrmann S, Cheung-Lung C, Brosseau J, Schwartz Y, Chassany O (2014). Electronic versus paper-based assessment of health-related quality of life specific to HIV disease: reliability study of the PROQOL-HIV questionnaire. J Med Internet Res.

[44] Hohwü L, Lyshol H, Gissler M, Jonsson SH, Petzold M, Obel C (2013). Web-based versus traditional paper questionnaires: a mixed-mode survey with a Nordic perspective. J Med Internet Res.

[45] (2006). World Health Organization.

[46] McHattie L, Cumming G, French T (2014). Transforming patient experience: health web science meets medicine 2.0. Med 2 0.

[47] Coons SJ, Gwaltney CJ, Hays RD, Lundy JJ, Sloan JA, Revicki DA, Lenderking WR, Cella D, Basch E (2009). Recommendations on evidence needed to support measurement equivalence between electronic and paper-based patient-reported outcome (PRO) measures: ISPOR ePRO Good Research Practices Task Force report. Value Health.

[48] Boers M, Brooks P, Strand CV, Tugwell P (1998). The OMERACT filter for outcome measures in rheumatology. J Rheumatol.

[49] Stableford S, Mettger W (2007). Plain language: a strategic response to the health literacy challenge. J Public Health Policy.

[50] Trudeau C (2016). Plain language in healthcare: what lawyers need to know about health literacy. Mich B J.

