# Supplementary material for: Using Plain Language and Adding Communication Technology to an Existing Health-Related Questionnaire to Help Generate Accurate Information: Qualitative Study
Source: J Med Internet Res. 2018 Apr 23;20(4):e140. doi: 10.2196/jmir.7940 (PMC5938598; doi:10.2196/jmir.7940)

Multimedia Appendix 1 An overview of the screenshots of the DTTSQ

1. 'Welcome'

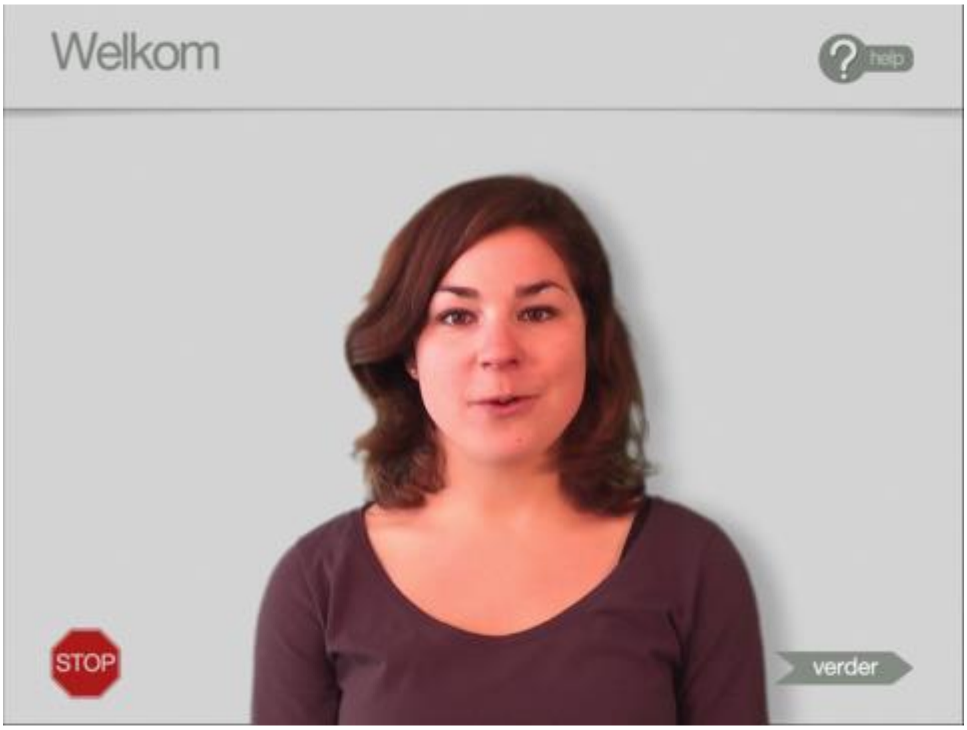

2. 'Pain'
"Do you have pain?"

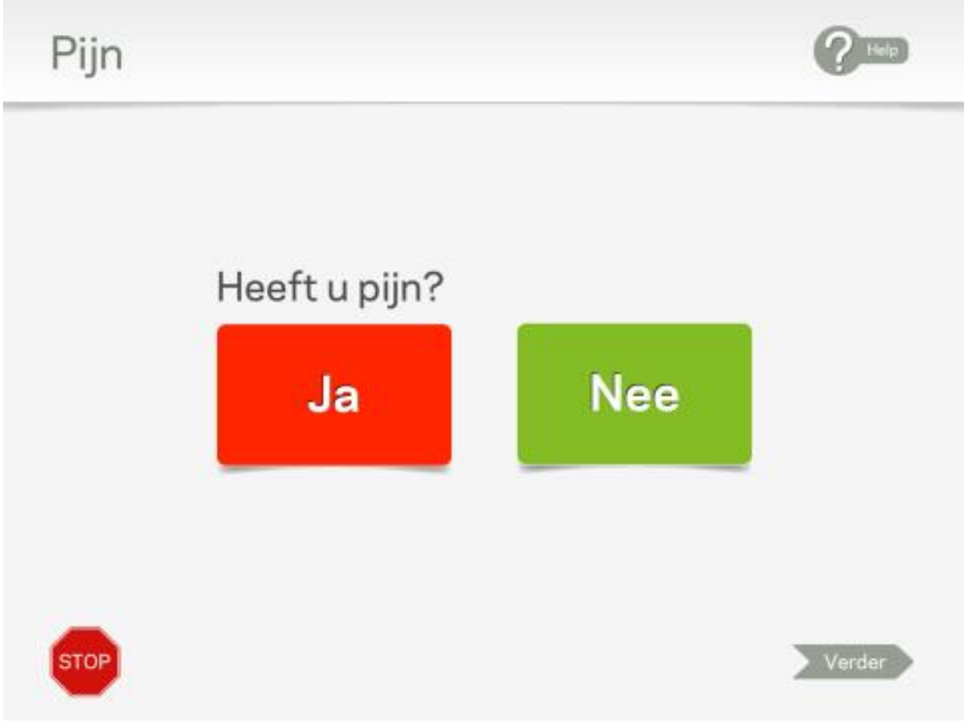

3. 'Location of the health problem'
"Tap on the location of your health problem. You can tap on multiple locations."

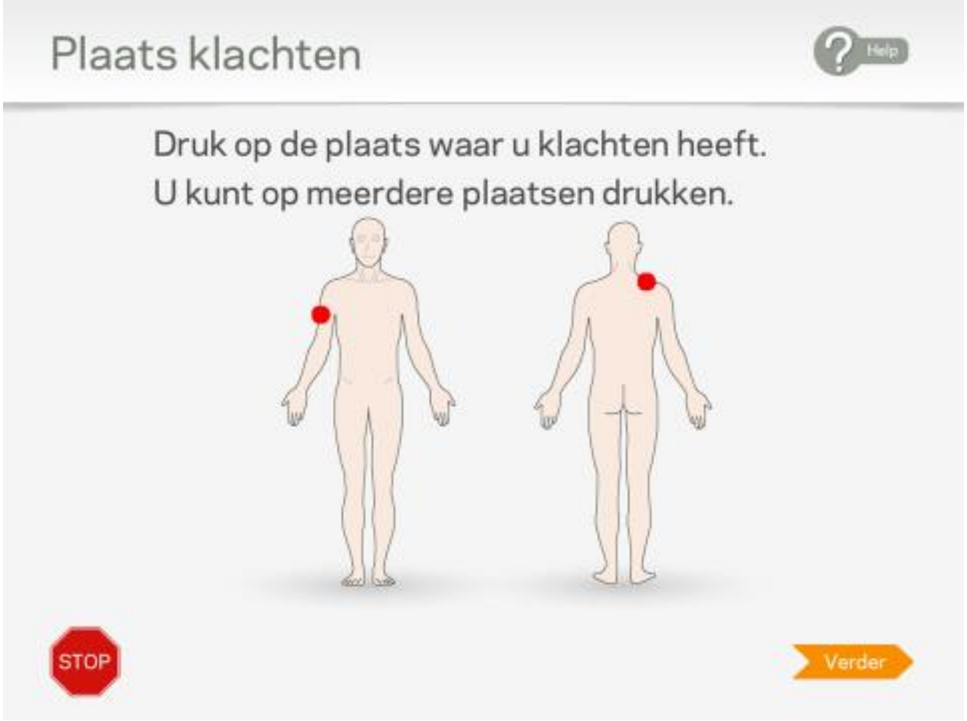

4. 'pain severity'
"This is the location of your pain.
Rate the severity of your pain on the scale below."

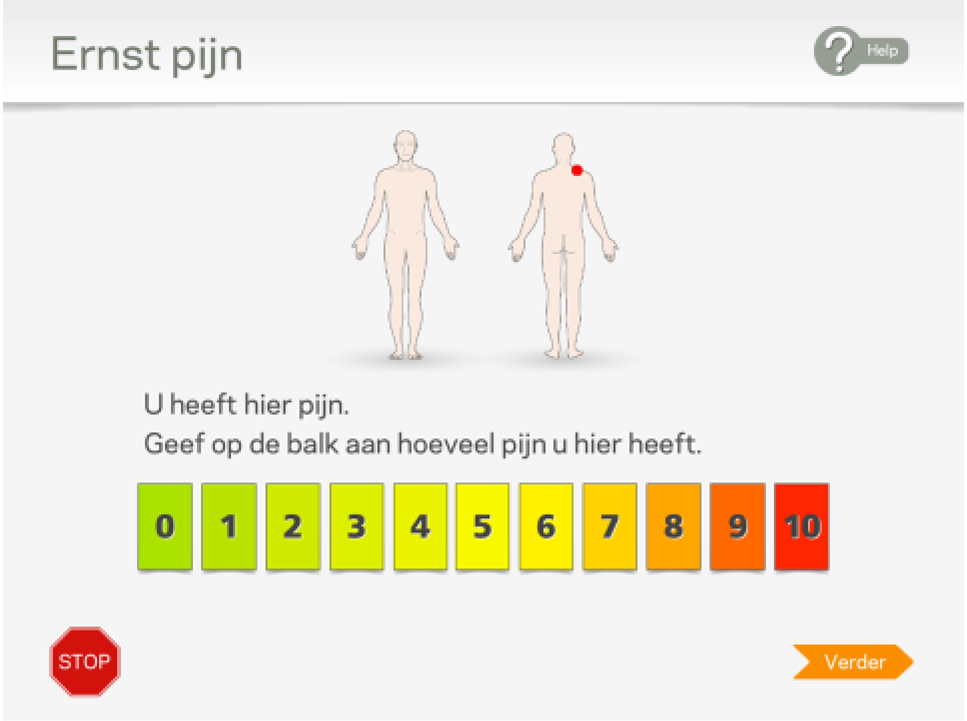

5. 'Overview location of the health problems'
"This is the location of your health problems."

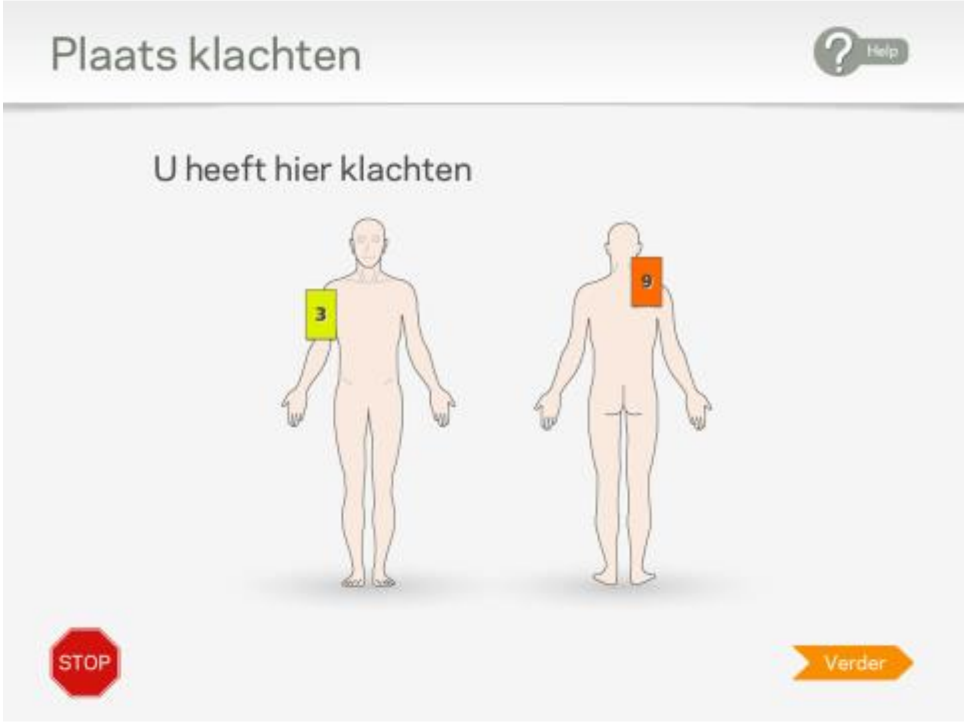

6. 'Activities'

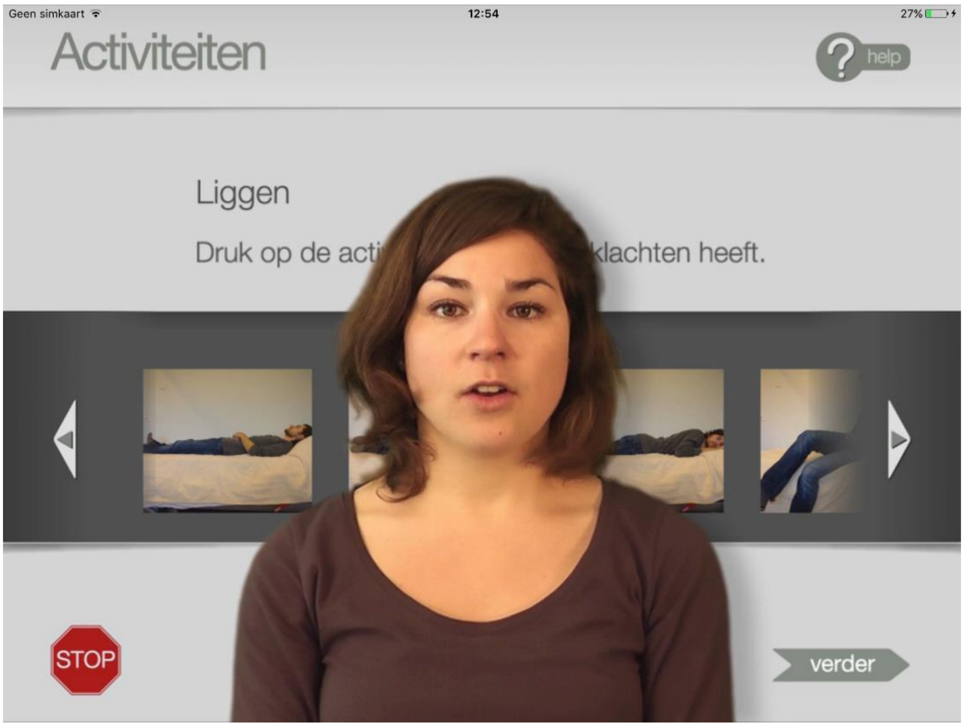

7. Activity 'lying'
"Select the activities in which you are impaired"

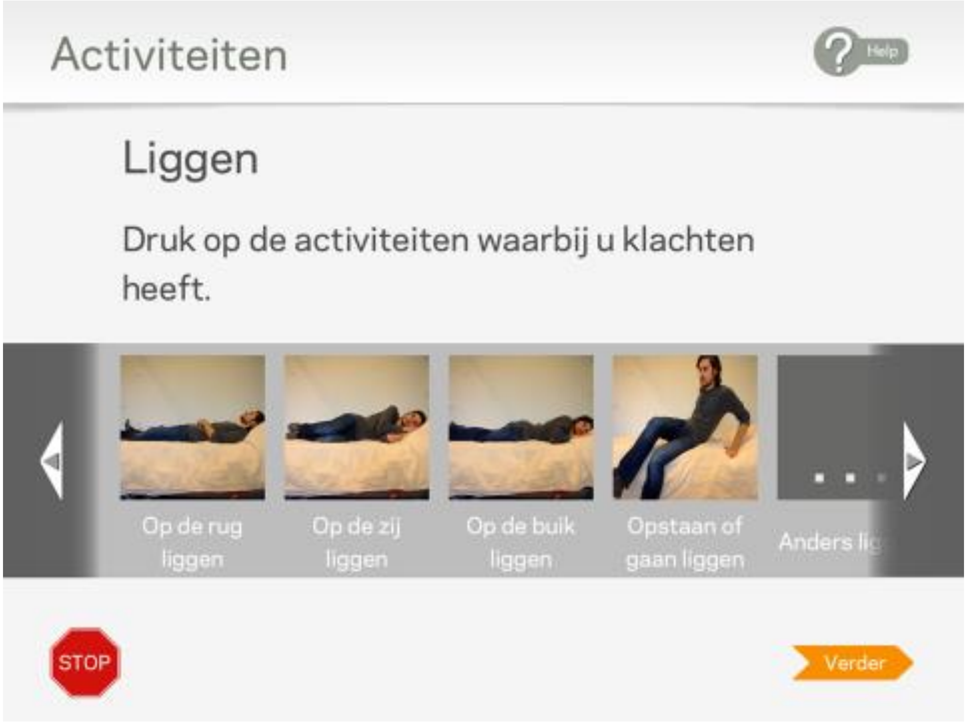

8. 'Overview activities'
"On this screen you see all the activities that you selected in previous screens. These are the activities in which you are impaired."

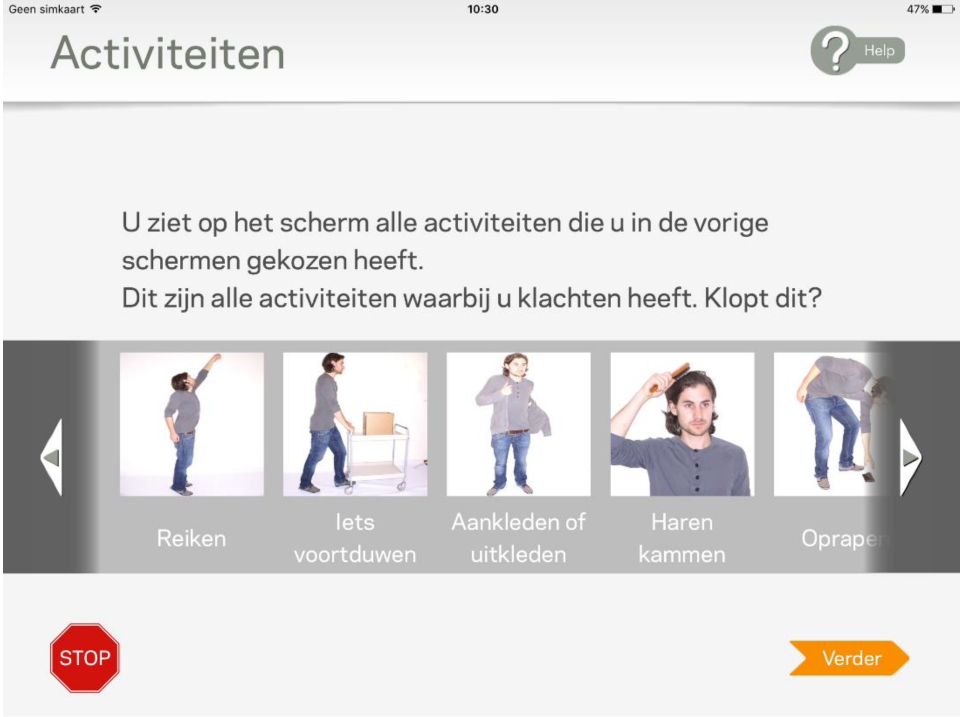

9. 'Most important activities'

"Select the three activities which are most important to you"

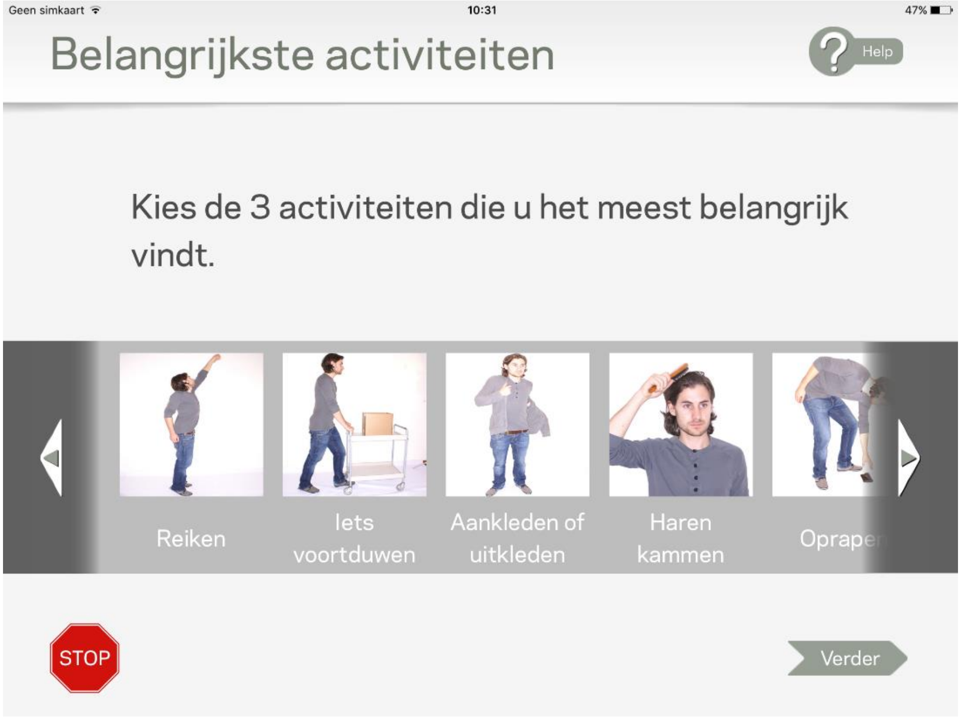

10. 'overview most important activities'

"You chose these three activities. Is this correct?"

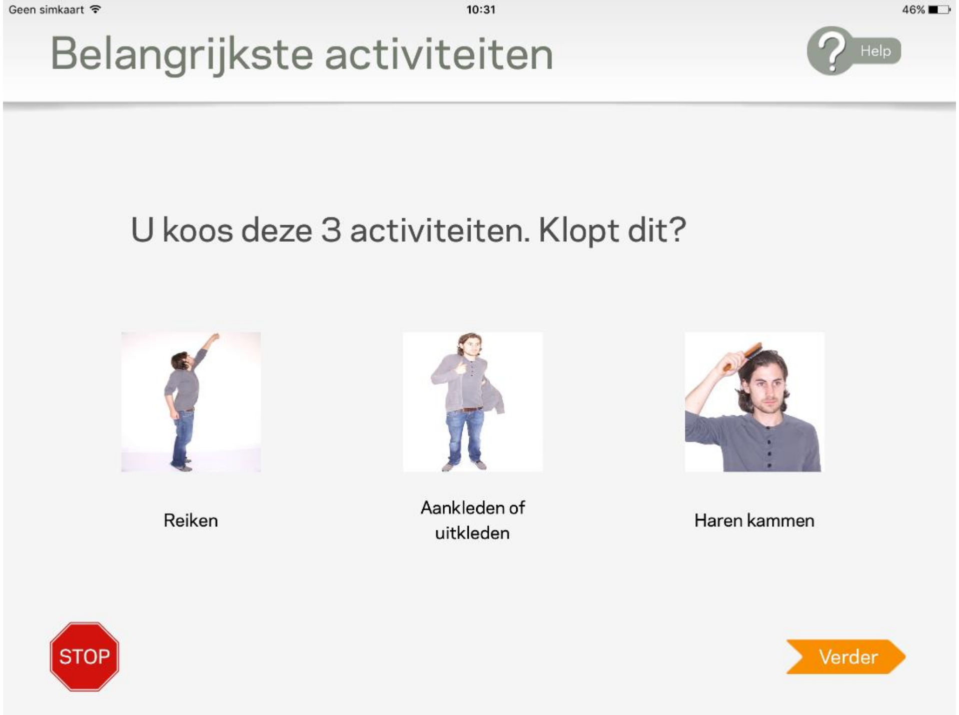

11. ‘Most important activity 1’

“Select the activity which is most important to you”

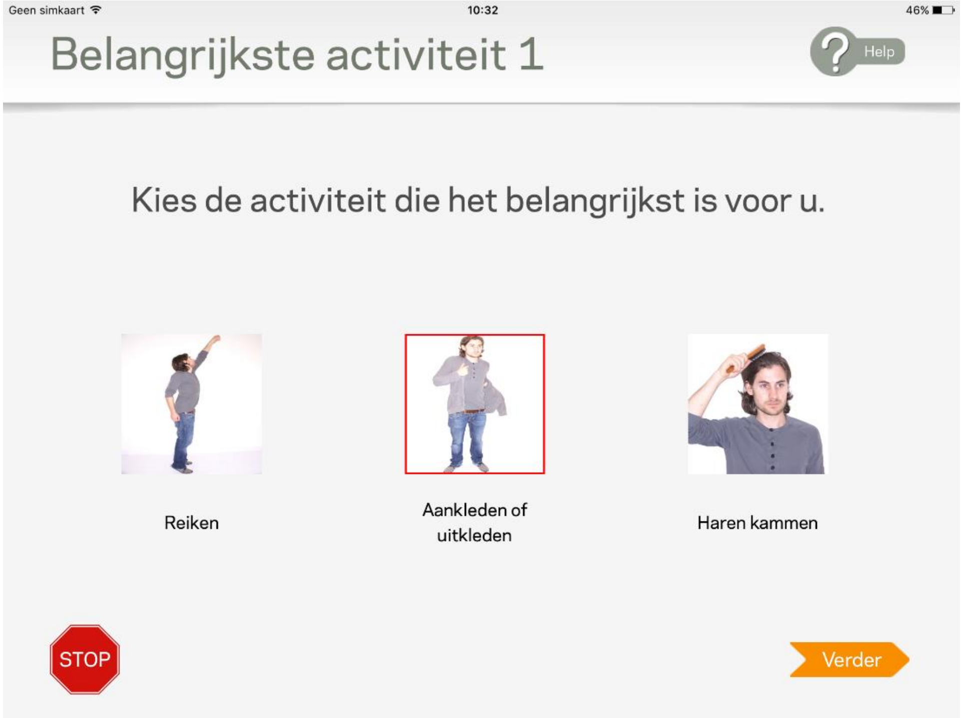

12. ‘Most important activity 2’

“Which of these two activities is still most important for you now?”

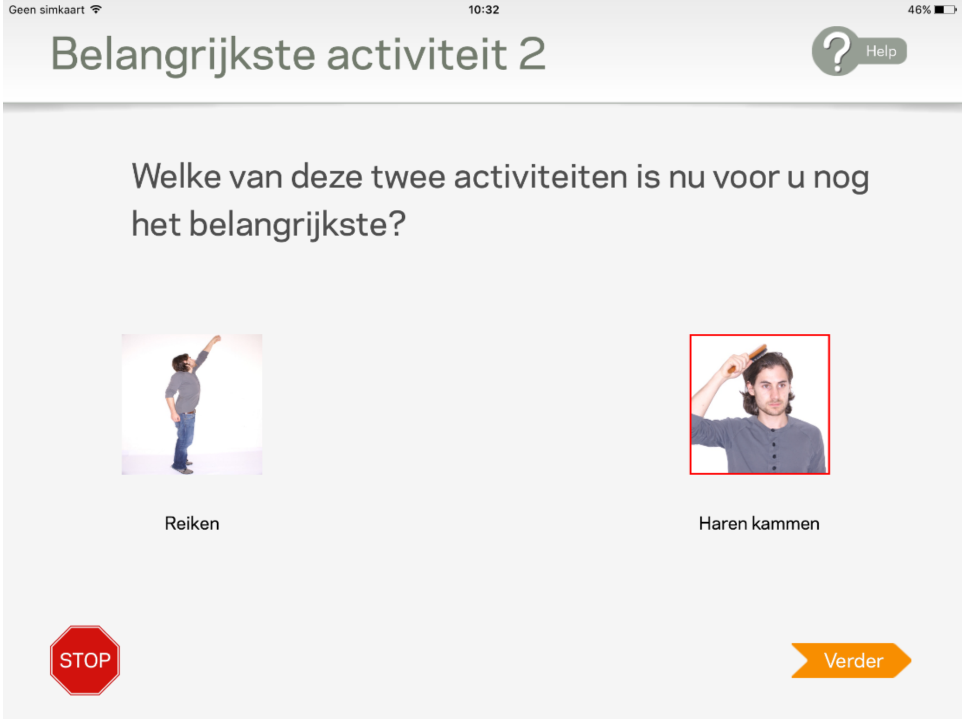

13. ‘Effort activity 1’

“Rate the effort it takes to carry out this activity“

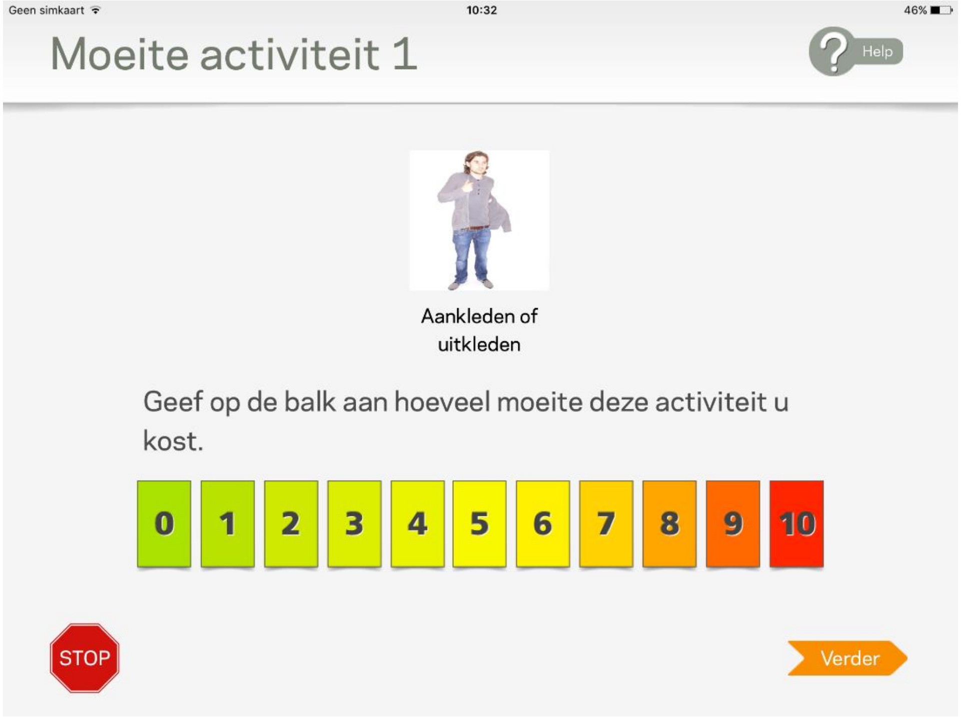

14. ‘overview most important activities and effort’

“On this screen you see the activities that are most important to you in order of most important to least important. Is this correct?”

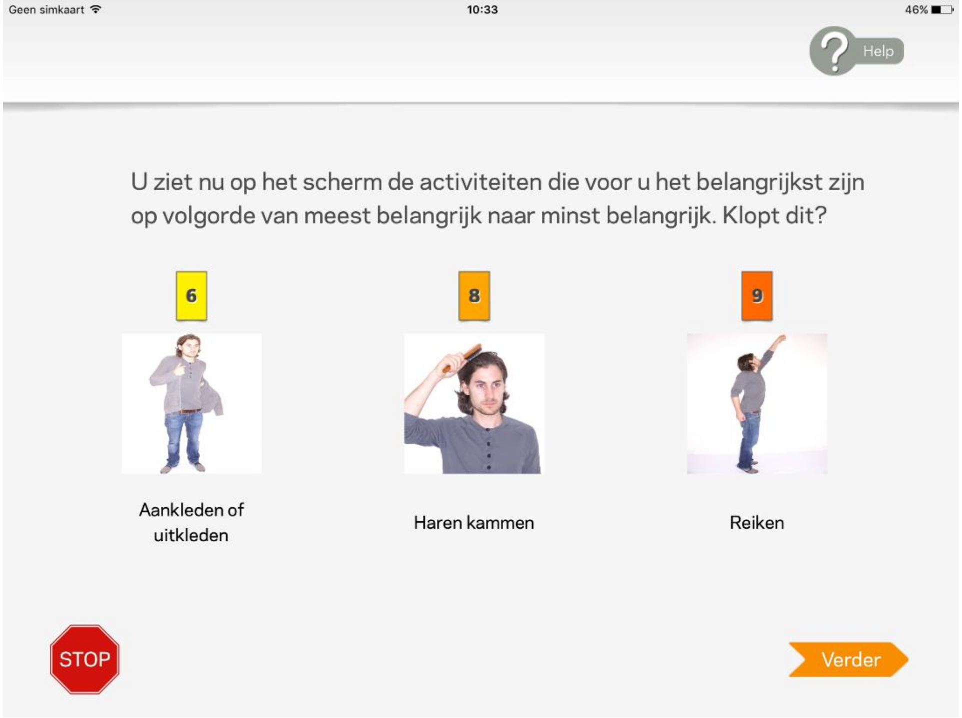

15. 'overview all outcomes of the questionnaire'

"On the screen you see an overview of all your answers you provided until now."

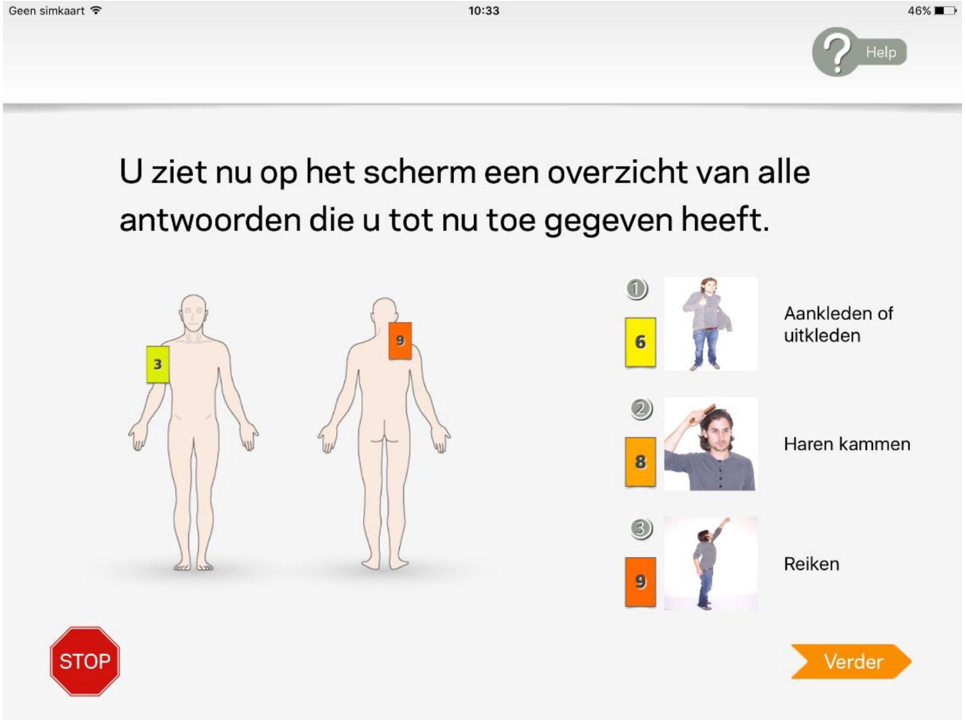

16. 'Thank you'

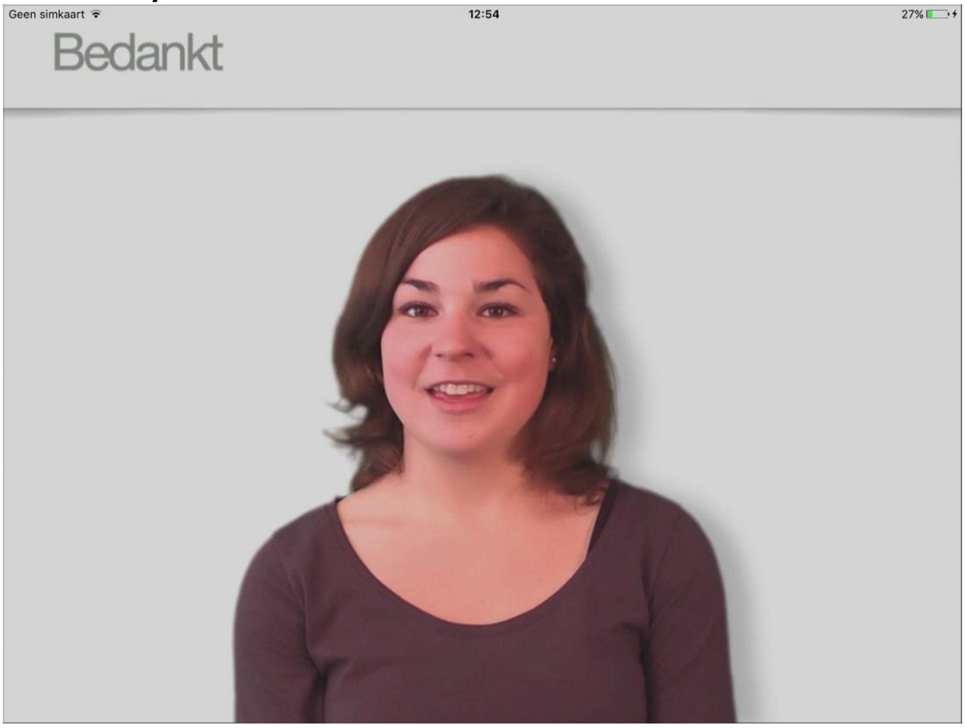

Supplement: Multimedia Appendix 1 [file jmir_v20i4e140_app1.pdf]
